# A β-catenin chromobody-based probe highlights endothelial maturation during vascular morphogenesis *in vivo*

**DOI:** 10.1242/dev.202122

**Published:** 2024-06-07

**Authors:** Sébastien Gauvrit, Shengnan Zhao, Ulrich Rothbauer, Didier Y. R. Stainier

**Affiliations:** ^1^ Department of Developmental Genetics, Max Planck Institute for Heart and Lung Research, 61231 Bad Nauheim, Germany; ^2^ Pharmaceutical Biotechnology, University of Tübingen, 72076 Tübingen, Germany; ^3^ Cluster of Excellence iFIT (EXC2180) ‘Image-Guided and Functionally Instructed Tumor Therapies’, University of Tübingen, 72076 Tübingen, Germany

**Keywords:** Nanobody, Chromobody, Live imaging, β-catenin, Vascular morphogenesis, Endothelium, Zebrafish

## Abstract

Visualization of protein dynamics is a crucial step in understanding cellular processes. Chromobodies, fluorescently labelled single-domain antibodies, have emerged as versatile probes for live cell imaging of endogenous proteins. However, how these chromobodies behave *in vivo* and how accurately they monitor tissue changes remain poorly explored. Here, we generated an endothelial-specific β-catenin chromobody-derived probe and analyzed its expression pattern during cardiovascular development in zebrafish. Using high-resolution confocal imaging, we show that the chromobody signal correlates with the localization of β-catenin in the nucleus and at cell-cell junctions, and thereby can be used to assess endothelial maturation. Loss of Cadherin 5 strongly affects the localization of the chromobody at the cell membrane, confirming the cadherin-based adherens junction role of β-catenin. Furthermore, using a genetic model to block blood flow, we observed that cell junctions are compromised in most endothelial cells but not in the endocardium, highlighting the heterogeneous response of the endothelium to the lack of blood flow. Overall, our data further expand the use of chromobodies for *in vivo* applications and illustrate their potential to monitor tissue morphogenesis at high resolution.

## Teaser

**Summary:** Generating a transgenic line based on an endothelial-specific β-catenin chromobody sheds light on the dynamics of endothelial maturation in vascular development.

## INTRODUCTION

The advancement of imaging tools has been instrumental in understanding developmental processes as well as cell biological mechanisms (Beis and Stainier, 2006; Cutrale et al., 2019). Among these tools, antibodies (IgGs) are key reagents to label and track proteins due to their high affinity and specificity. However, because of their large size and complex structures, IgGs cannot be applied within living cells. More recently, nanobodies, single-domain antibodies from camelids, have emerged as new tools to study protein dynamics and function (Aguilar et al., 2019; Wagner and Rothbauer, 2020). Nanobodies provide additional useful features compared with conventional antibodies, such as high stability and smaller size, thereby allowing deeper tissue penetration. To visualize the dynamics of intracellular proteins, nanobodies can be genetically fused with fluorescent proteins and introduced as short intracellular functional transgenes (so-called chromobodies), thereby expanding their utility.

The discovery of a chromobody against GFP has allowed the tracking of GFP-tagged proteins, and different strategies have also used it as a scaffold to control the level of expression of tagged proteins or relocalize them to different cell compartments (Rothbauer et al., 2006; Harmansa et al., 2017; Yamaguchi et al., 2019; Juan et al., 2024). It has also been documented that chromobodies against endogenous proteins, including the major cytoskeletal component actin and the cell cycle marker PCNA, can operate as ready-to-use probes for *in vivo* tracking in zebrafish (Panza et al., 2015). To date, however, only a few studies have used chromobodies *in vivo* to monitor tissue morphogenesis and follow dynamic changes. In this context, the cardiovascular system is ideal to investigate how nanobodies and chromobodies can reveal physiological changes in tissue. Indeed, endothelial cells, which line blood and lymphatic vessels, are exposed to different types of mechanical forces on their inner/luminal (e.g. blood flow triggering shear stress and laminar stress) and outer/abluminal (e.g. tissue growth, interactions with mural cells, extracellular matrix stiffness) sides (Collins and Stainier, 2016; Gordon et al., 2020). Specifically, the intricate balance between cell differentiation and tissue growth is particularly evident for the endocardium, as it is exposed to increased and altered hemodynamic forces during development. In the absence, or disruption, of these forces, cardiovascular development is abnormal, suggesting that the whole tissue must sense these variations and respond appropriately.

Here, we used a β-catenin chromobody labelled with EGFP (Traenkle et al., 2015) to generate an endothelial-specific chromobody-based β-catenin probe in zebrafish. β-Catenin is a highly conserved multifunctional protein that is essential for cadherin-based adherens junctions and is also the crucial nuclear effector of Wnt/β-catenin signalling (also known as canonical Wnt signalling) (Valenta et al., 2012). Using this optical probe in combination with high-resolution imaging and time-lapse microscopy, we show that the β-catenin chromobody displays dynamic localization in different subcellular compartments across different developmental stages, mainly in the nucleus and at the cell membrane. Furthermore, we observed a more dominant β-catenin chromobody localization at endothelial cell membranes at later stages, which can be used to assess the maturation of endothelial cell junctions.

By crossing this new chromobody transgenic line with different mutational backgrounds, we show how the physiological state of the vascular system can be assessed. For example, by comparing different vascular beds in response to the lack of blood flow, we observed that the endocardium maintains its cell-cell junctions compared with the trunk endothelium, revealing endothelial heterogeneity. In summary, our data show how this new chromobody-based probe helps to gain further insight into vascular development and expands our toolkit to better understand how vascular malformations arise in a specific vessel type or vascular bed.

## RESULTS AND DISCUSSION

**Fig. 1.**
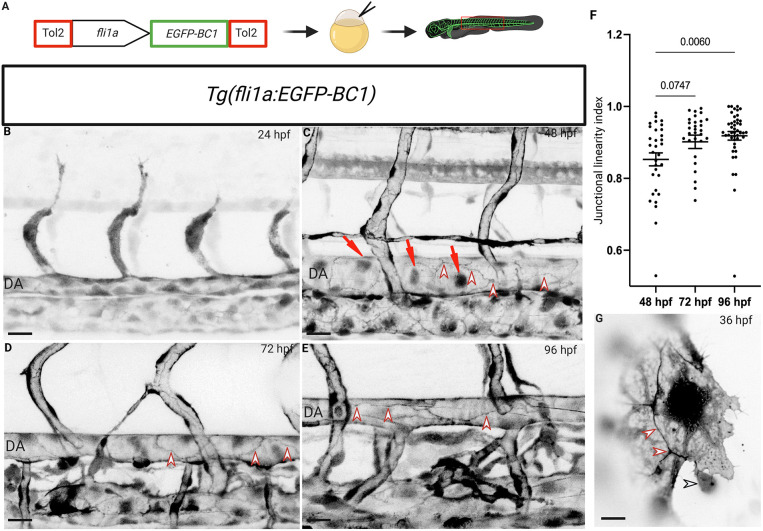
**The endothelial-specific β-catenin chromobody exhibits expression in endothelial nuclei and cell-cell junctions and can be used to monitor junctional maturation during zebrafish development.** (A) Schematic of the *fli1a:EGFP-BC1* construct. (B-E) Trunk vasculature of *Tg(fli1a:EGFP-BC1)* zebrafish from 24 to 96 hpf. Chromobody expression localizes to endothelial nuclei (red arrows) and at cell membranes (red arrowheads). (F) Junctional linearity index at 48 (*n*=32 junctions from six embryos), 72 (*n*=30 junctions from six larvae) and 96 (*n*=44 junctions from seven larvae) hpf. Data are mean±s.d.; *P*-value calculated using an unpaired two-tailed Student's *t*-test. (G) Single plane spinning disc confocal image of a single endothelial cell during CCV formation at 36 hpf. The β-catenin chromobody exhibits a dynamic localization (see Movie 1), especially at endothelial cell-cell junctions; interactions of red blood cells with endothelial cells can also be observed (red arrowheads point to endothelial cell-cell junctions; black arrowhead points to a red blood cell interacting with an endothelial cell). Scale bars: 40 μm (B-E); 10 μm (G).

To track β-catenin in zebrafish, we decided to use a previously characterized chromobody directed against β-catenin (Traenkle et al., 2015). Notably, the BC1 chromobody binds β-catenin without interfering with its transcriptional activity (Traenkle et al., 2015). We expressed the BC1 chromobody (fused with EGFP) specifically in endothelial cells using the zebrafish *fli1a* promoter (Lawson and Weinstein, 2002) (Fig. 1A). For *in vivo* applications, a stable transgenic line, *Tg(fli1a:EGFP-BC1)*, was generated, in which embryos appeared to be unaffected; fish were raised to adulthood, suggesting that constitutive expression of a chromobody is well tolerated, as previously shown (Panza et al., 2015). To characterize this new transgenic line, we first focused on the trunk vasculature at different developmental stages, as it is the best characterized vascular bed during development (Isogai et al., 2003). At 24 hours post-fertilization (hpf), the localization of the β-catenin chromobody was primarily cytosolic and nuclear (Fig. 1B). From 55 hpf, the expression pattern changes as cell junctions become more prominently labelled while nuclear expression remains present (Fig. 1C-E). Comparing the expression pattern of the β-catenin chromobody with that of *Tg(fli1a:*GFP*)* further confirmed the difference from the expression of EGFP alone (Fig. S1A-B′). Next, we measured junction linearity at different stages to determine whether the *fli1a:EGFP-BC1* transgene could be used to visualize endothelial maturation. We observed that the junction linearity increases from 55 to 96 hpf (Fig. 1F). In addition, images revealed that early endothelial cell junctions are irregular and immature compared with junctions at later stages, as previously shown (Lagendijk et al., 2017). It has been shown that the β-catenin chromobody, when expressed in Hela cells, labels only the cytoplasmic form of β-catenin but does not localize to cell-cell junctions (Traenkle et al., 2015). However, these experiments were carried out in cells in culture, which do not display the organization of epithelial-like endothelial cells. Moreover, these images were taken using an epifluorescence microscope that does not have the same resolution as a confocal microscope.

To better understand the dynamics of the chromobody, we used high-speed spinning-disc microscopy to image endothelial cell migration during common cardinal vein (CCV) development. Starting from 20 hpf, the endothelial cells that will form the CCV migrate away from the midline and start to align as a monolayer on top of the yolk syncytial layer (YSL) (Helker et al., 2013). This process provides a unique opportunity to image a monolayer of endothelial cells with a migratory front and to use high-resolution microscopy thanks to its location on top of the YSL. At 36 hpf, we observed that chromobody localization was highly dynamic, especially at endothelial cell-cell junctions (Fig. 1G; Movie 1). We could also observe the interaction of circulating cells with endothelial cells during their migration (Fig. 1G; Movie 1), as previously shown (Helker et al., 2013).

**Fig. 2.**
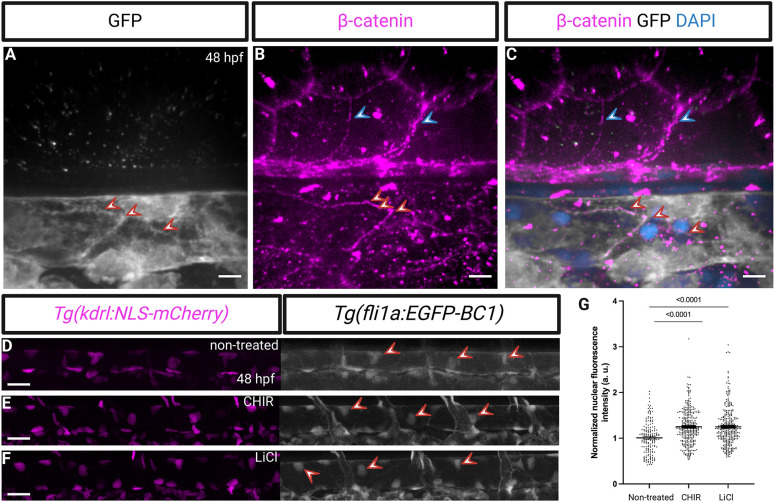
**β-Catenin chromobody expression in endothelial nuclei.** (A-C) Confocal images of immunostaining (GFP, white; β-catenin, magenta) and counterstaining for DNA (DAPI, blue) of *Tg(fli1a:EGFP-BC1)* embryos at 48 hpf. β-Catenin immunostaining coincides with the chromobody localization at the endothelial cell-cell junctions (red arrowheads point to endothelial cell-cell junctions, blue arrowheads to non-endothelial β-catenin immunostaining). (D-G) Lateral views and quantification of fluorescence intensity of the nuclear signal in dorsal aorta endothelial cells of 48 hpf *Tg(fli1a:EGFP-BC1); Tg(kdrl:NLS-mCherry)* embryos after exposure to the indicated GSK3β inhibitor starting at 24 hpf (non-treated, *n*=166 nuclei from six embryos; CHIR, *n*=305 nuclei from 12 embryos; LiCl, *n*=311 nuclei from 12 embryos). Treatment with either GSK3β inhibitor increases the nuclear signal of the *Tg(fli1a:EGFP-BC1)* chromobody. Data are mean±s.d. *P<*0.0001 calculated using a one-way ANOVA (post hoc Tukey's test). Red arrowheads point to endothelial nuclei. Scale bars: 40 μm (A-C), 10 μm (D-F).

To further determine the localization of the chromobody in endothelial cells, we performed an immunostaining for β-catenin on 48 hpf *Tg(fli1a:EGFP-BC1)* zebrafish embryos (Fig. 2A-C). Notably, the chromobody signal colocalized with β-catenin immunostaining at the endothelial cell-cell junctions, but we did not observe β-catenin immunostaining in the nucleus of endothelial cells (or of other cell types). The absence of nuclear β-catenin immunostaining could be because the antibody used recognizes the N-terminally phosphorylated form of β-catenin, which has not been observed in the nucleus (Valenta et al., 2012). In addition, the BC1 chromobody has been shown to recognize a discontinuous epitope within the N-terminal domain [amino acids (aa) 1-119] of β-catenin (Traenkle et al., 2015) that overlaps with the epitope for the antibody (around aa 37) and, thus, binding of the BC1 chromobody to β-catenin might prevent the binding of the antibody. For these reasons, we then decided to use another antibody, one that recognizes β-catenin at its C terminus (aa 768-781) and that has been recently reported to recognize nuclear and cell junction-localized β-catenin in the zebrafish dorsal blastula (Yan et al., 2018). We injected *EGFP-BC1* mRNA at the one-cell stage and collected embryos at the 512-cell stage for immunostaining. We observed in dorsal blastomeres that the chromobody signal colocalized with β-catenin immunostaining at cell-cell junctions as well as in the nucleus (Fig. S2A-C).

To investigate whether manipulating the Wnt/β-catenin pathway induces a change in the chromobody localization, we used two glycogen synthase kinase 3 β (GSK3β) inhibitors, CHIR and LiCl, which inhibit the cytoplasmic degradation of β-catenin, thereby increasing its accumulation and nuclear translocation. We treated *Tg(fli1a:EGFP-BC1);Tg(kdrl:NLS-mCherry)* embryos with the respective compounds from 24 to 48 hpf and imaged the dorsal aorta. To quantify the nuclear fluorescence intensity, we delineated the nuclei and normalized the data using *Tg(kdrl:*NLS-mCherry*)* expression. Quantitative image analysis showed an increased nuclear signal of the *Tg(fli1a:EGFP-BC1)* chromobody after GSK3β inhibitor treatment, suggesting that it could potentially be used to investigate β-catenin translocation *in vivo* (Fig. 2D-G). This result correlates with data obtained using another zebrafish transgenic line designed to report β-catenin transcriptional activity in endothelial cells (Kashiwada et al., 2015).

**Fig. 3.**
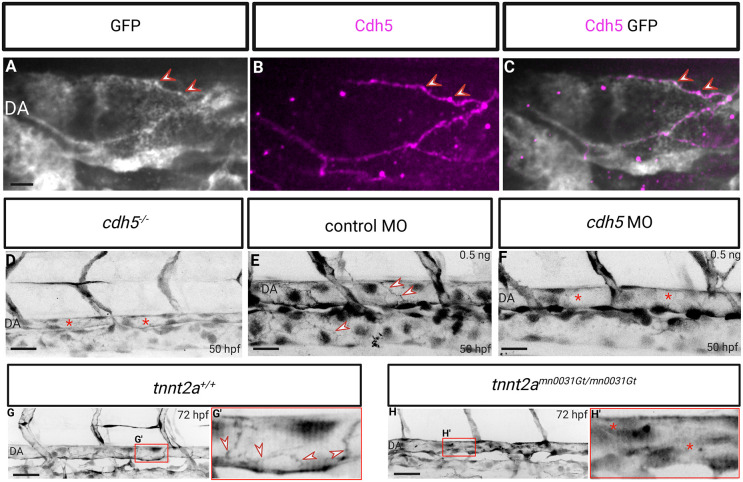
**Membrane localization of the endothelial-specific β-catenin chromobody is dependent on Cdh5 expression and blood flow.** (A-C) Confocal images of immunostaining (GFP, white; Cdh5, magenta) of *Tg(fli1a:EGFP-BC1)* embryos at 48 hpf. Cdh5 co-localizes with the chromobody at the endothelial cell-cell junctions (red arrowheads). (D) Trunk vasculature of *Tg(fli1a:EGFP-BC1)*; *cdh5^−/−^* embryos at 50 hpf. *cdh5* mutants mostly lack blood flow and display a lack of localization of the β-catenin chromobody at endothelial cell-cell junctions (asterisks indicate a collapsed dorsal aorta due to the lack of blood flow). (E,F) Trunk vasculature of 50 hpf *Tg(fli1a:EGFP-BC1)* embryos injected at the one-cell stage with 0.5 ng control MO (E) or 0.5 ng *cdh5* MO (F). *cdh5* morphants display a lack of localization of the β-catenin chromobody at endothelial cell-cell junctions while blood flow is not affected (arrowheads point to β-catenin chromobody localization at endothelial cell-cell junctions; asterisks indicate a lack of localization of the β-catenin chromobody at endothelial cell-cell junctions). (G,H) Trunk vasculature of *Tg(fli1a:EGFP-BC1)*; *tnnt2a^+/+^* and *Tg(fli1a:EGFP-BC1)*; *tnnt2a^−/−^* (*tnnt2a*^mn0031Gt/mn0031Gt^) larvae at 72 hpf. *tnnt2a* mutants display a lack of blood flow, and the localization of the β-catenin chromobody at endothelial cell-cell junctions is missing. (G′,H′) High-magnification images of boxed areas in G and H, showing the dorsal aorta. Arrowheads point to β-catenin chromobody localization at endothelial cell-cell junctions; asterisks indicate a lack of localization of the β-catenin chromobody at endothelial cell-cell junctions. Scale bars: 40 μm.

It is well established that, in endothelial cells, β-catenin interacts directly with Cadherin 5 (Cdh5, also known as VE-Cadherin) (Carmeliet et al., 1999). To investigate whether the chromobody signal colocalizes with Cdh5 at endothelial cell-cell junctions, *Tg(fli1a:EGFP-BC1)* zebrafish embryos were immunostained for Cdh5 at 48 hpf. The observed overlap of chromobody and antibody signals at endothelial cell-cell junctions indicates that the chromobody and Cdh5 indeed colocalize at the membrane (Fig. 3A-C). Next, we further investigated this association by crossing the *Tg(fli1a:EGFP-BC1)* line with the *cdh5* mutant line (Sauteur et al., 2014), and observed that chromobody expression was not present at endothelial cell-cell junctions in *cdh5* mutants at 50 hpf (Fig. 3D). However, as *cdh5* mutants display a strong cardiovascular phenotype with no blood flow (Sauteur et al., 2014), we decided to use morpholinos (MO). Indeed, previous studies have shown that injecting 0.5 ng of *cdh5* morpholino was able to knock down Cdh5 expression without severely affecting blood flow (Bornhorst et al., 2019). By following this protocol, there was a substantial reduction of the β-catenin chromobody localization at endothelial cell-cell junctions compared with control morpholino injected embryos but blood flow was not obviously affected (Fig. 3E,F), indicating that decreased expression of Cdh5 reduces the localization of the β-catenin chromobody to the membrane. To pursue this line of investigation further, we decided to use another well-known model to modulate blood flow, namely the *tnnt2a* mutant zebrafish, which lacks cardiac contraction (Sehnert et al., 2002). We imaged *tnnt2a* mutants in the β-catenin chromobody line background and observed that, as with *cdh5* mutants, the endothelial cell-cell junction signal was not observed in the absence of blood flow (Fig. 3G-H′). To determine how the β-catenin chromobody behaves in endothelial cells during an acute loss of blood flow, we performed time-lapse microscopy after treatment with tricaine, which immediately stops cardiac contraction (Fig. S3). We observed that acute loss of blood flow did not appear to alter the localization of the β-catenin chromobody signal at endothelial membranes. Similarly, acute loss of blood flow did not affect junctional linearity after 90 min of treatment, confirming previous observations using a Cdh5 tension sensor (Lagendijk et al., 2017).

To further test the necessity of blood flow for the endothelial cell junction localization of the β-catenin chromobody, we decided to rescue the *tnnt2a* mutant phenotype using an allele in which a stop cassette can be removed by Cre activation (Clark et al., 2011). Thus, we combined this allele with the *Tg(cryaa:DsRed,-5.1myl7:CreER)* line, which can be activated using tamoxifen (Kikuchi et al., 2010), thereby controlling the timing of cardiac contractility and blood flow restoration (Fig. S4A). We added tamoxifen starting at 32 hpf and subsequently observed endothelial cell junction localization of the β-catenin chromobody in comparison with the non-rescued mutant embryo (Fig. S4B-E), even though the rescued mutants exhibited pericardial oedema and only a partial rescue of blood flow (Fig. S4D; Movie 2).

**Fig. 4.**
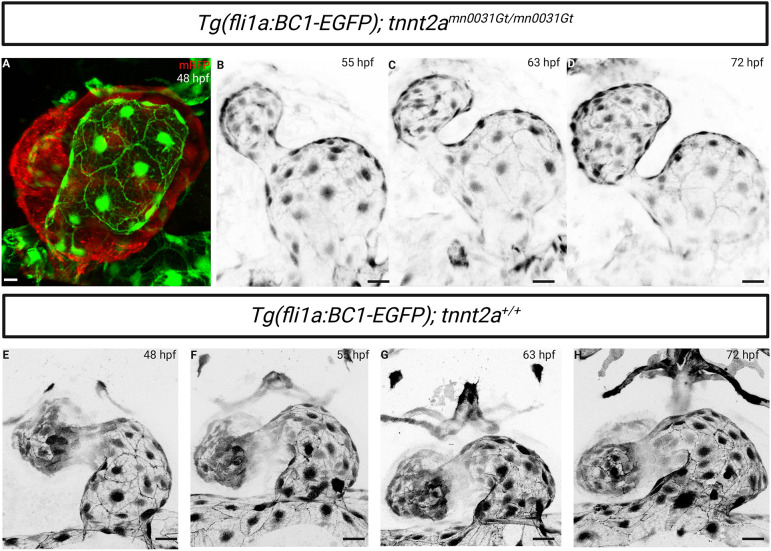
**Endocardial expression of the endothelial-specific β-catenin chromobody in *tnnt2a* mutants.** (A) Confocal imaging of a 48 hpf *Tg(fli1a:EGFP-BC1); tnnt2a^−/−^* atrium; mRFP expression corresponds to the *tnnt2a* gene trap expression. The endothelial-specific β-catenin chromobody is localized at endocardial cell-cell junctions in *tnnt2a^−/−^*. (B-D) Confocal time-lapse images of a *Tg(fli1a:EGFP-BC1); tnnt2^−/−^* atrium at 55, 63 and 72 hpf from Movie 3. Localization of the endothelial-specific β-catenin chromobody is maintained at endocardial cell-cell junctions despite the lack of blood flow. (E-H) Confocal time-lapse images of a *Tg(fli1a:EGFP-BC1); tnnt2a^+/+^* atrium at 48, 55, 63 and 72 hpf. Scale bars: 20 μm.

The response of endothelial cells to blood flow differs depending on their localization, and the molecular signature of every vascular bed is different (Cleuren et al., 2019). The developing endocardium is of particular interest due to its exposure to increased and altered hemodynamic forces during cardiac morphogenesis (Collins and Stainier, 2016). Hence, we examined the behaviour of the β-catenin chromobody in the endocardium in *tnnt2a* mutants. Surprisingly, the *tnnt2a* mutant endocardium displayed junctional localization of the β-catenin chromobody (Fig. 4) despite the lack of blood circulation, which strongly affects the trunk vasculature (Fig. 3). Time-lapse imaging from 55 to 72 hpf further showed a continuous junctional localization of the β-catenin chromobody in the endocardial cells (Movie 3). This difference in junctional localization of the β-catenin chromobody in the endocardium versus endothelium of *tnnt2a* mutants might be explained by the strong inter-layer communication within the developing heart, especially the increasing tensile forces between the myocardium and endocardium (Bornhorst et al., 2019; Priya et al., 2020; Qi et al., 2022) and/or the strong adhesive forces between endocardial cells during cardiac chamber expansion (Mellman et al., 2012).

In summary, the endothelial β-catenin chromobody reporter presented here expands the imaging toolkit available to investigate vascular development. It also provides a powerful tool to further investigate various vascular phenotypes including how vascular malformations arise in a specific vessel type or vascular bed. Considering that new nanobodies and chromobodies against numerous antigens can now be efficiently generated *in vitro* from synthetic libraries (McMahon et al., 2018), the portfolio of new probes will continue to expand, resulting in new opportunities to track protein localization not only in cells in culture but also *in vivo*. We anticipate that these new probes will have a major impact on the understanding of cell and developmental biology.

## MATERIALS AND METHODS

### Zebrafish

Zebrafish husbandry was performed in accordance with institutional (Max-Planck-Gesellschaft zur Förderung der Wissenschaften) and national (German) ethical and animal welfare regulations. Larvae were raised under standard conditions. Adult zebrafish were maintained in 3.5 l tanks at a stock density of 10 zebrafish/l with the following parameters: water temperature, 27-27.5°C; 14 h light/10 h dark cycle; pH 7.0-7.5; conductivity, 750-800 µS/cm. Zebrafish were fed 3-5 times a day, depending on age, with granular and live food (*Artemia salina*). Health monitoring was performed at least once a year. All embryos and larvae used in this study were raised at 28°C. All procedures performed on animals conform to the guidelines from Directive 2010/63/EU of the European Parliament on the protection of animals used for scientific purposes and were approved by the Animal Protection Committee (Tierschutzkommission) of the Regierungspräsidium Darmstadt (reference: B2/1218). We used the following zebrafish lines: *Tg(fli1a:EGFP)^y1^* (Lawson and Weinstein, 2002), *tnnt2a^mn0031Gt^* (Clark et al., 2011), *Tg(cryaa:DsRed,-5.1myl7:CreER)^pd10^* (Kikuchi et al., 2010), *cdh5^ubs8^* (Sauteur et al., 2014) and *Tg(kdrl:NLS-mcherry)^is4^* (Wang et al., 2010).

### Transgenesis

To generate the *Tg(fli1a:EGFP-BC1)^bns320^* line, *EGFP-BC1* was inserted into a *fli1a* promoter plasmid (Lawson and Weinstein, 2002). Then 25 pg of Tol2 transposase mRNA and 25 pg of *pTol2-fli1a:EGFP-BC1* DNA were co-injected at the one-cell stage. Potential founders were screened by fluorescence in the F1 generation.

### Live imaging

Images were acquired using a LEICA TCS SP8 inverted microscope equipped with a 40×/1.1 objective or a Zeiss Cell Observer SD inverted microscope equipped with a 63× objective. Image acquisition was performed after embryo anaesthesia with 0.01% tricaine and immobilization in 0.5% low-melting agarose in glass bottom Petri dishes (MatTek corporation). For time-lapse imaging, images were acquired using a Zeiss Cell Observer SD microscope.

### Junctional linearity quantification

Junctional linearity was quantified using Fiji (Schindelin et al., 2012) as previously described (Lagendijk et al., 2017). Maximum intensity projections of the EGFP signal from the *Tg(fli1a:EGFP-BC1)^bns320^* line were generated and junction linearity from endothelial cells of the dorsal aorta were calculated by dividing the distance between the junctional vertices over the length of the junction.

### Morpholino injections

Knockdown experiments were performed by injection of the following MO (Gene Tools): *cdh5*, 5′-TTTACAAGACCGTCTACCTTTCCAA-3′ (Mitchell et al., 2010); control, 5′-CCTCTTACCTCAGTTACAATTTATA-3′. We injected 0.5 ng of control or *cdh5* MO into one-cell stage embryos.

### Immunostaining

Embryos were fixed at 48 hpf using PEM [3% paraformaldehyde (PFA), 100 mM Pipes, 1 mM MgSO_4_ and 2 mM EGTA in distilled water and adjusted to pH 7.4] for 3 h at room temperature (RT). Embryos were washed with PBS/0.1% Tween (PBST) three times then permeabilized using proteinase K (10 μg/ml) in PBST for 25 min. Embryos were rinsed with PBDT [1× PBS, 1% bovine serum albumin (BSA), 1% dimethyl sulfoxide, and 0.5% Triton X-100], followed by incubation in blocking buffer (1× PBST, 10% sheep serum, 0.8% Triton X-100 and 1% BSA) for 2 h at RT. Samples were then incubated with primary antibodies in incubation buffer (1× PBST, 1% sheep serum, 0.8% Triton X-100 and 1% BSA) overnight at 4°C. After rinsing with PBDT, embryos were incubated with secondary antibodies in incubation buffer for 3 h at RT. Samples were stained with 4′,6-diamidino-2-phenylindole (DAPI, 1:1000, 10 mg/ml stock, Sigma-Aldrich) in PBST for 10 min, washed five times with PBST and then mounted in 2% low-melting agarose (Sigma-Aldrich) for imaging. Imaging was carried out using a Zeiss Cell Observer SD microscope equipped with a 63×/1.3 objective. Primary antibodies used for immunostaining were: anti-β-catenin (rabbit, 9562S, Cell Signaling Technology, 1:50) recognizing the N terminus, anti-β-catenin (rabbit, C2206, Sigma-Aldrich, 1:50) recognizing the C terminus, anti-Cdh5 (rabbit, AS-55715, AnaSpec, 1:50) and anti-GFP (chicken, GFP-1020, Aveslabs, 1:200). Secondary antibodies used were Alexa Fluor 568-conjugated goat anti-chicken secondary antibodies (A11041, Thermo Fisher Scientific, 1:1000) and Alexa Fluor 647-conjugated goat anti-rabbit secondary antibodies (A21244, Thermo Fisher Scientific, 1:1000).

### mRNA injections

*EGFP-BC1* was amplified using the following primers: F: 5′-ATTTAGGTGACACTATAGAGGTTTAGTGAACCG-3′; R:5′-TAAGATACATTGATGAGTTTGGACAAACCACAAC-3′. *EGFP-BC1* mRNA was synthesized using a mMESSAGE mMACHINE SP6 kit (Life Technologies) and purified using an RNA Clean and Concentrator Kit (Zymo Research). *EGFP-BC1* mRNA was injected at 200 pg per embryo at the one-cell stage and collected at the 512-cell stage for immunostaining.

### Pharmacological treatment

LiCl (100 mM) and CHIR (5 µM) were used as chemical inhibitors. Embryos were treated from 24 to 48 hpf. LiCl was diluted in egg water. CHIR was prepared in 100% DMSO and diluted to the indicated concentration with egg water. As a control, volume-matched DMSO solutions in egg water were used. The nuclear area was determined using *Tg(kdrl:*NLS-mcherry*)* expression. Quantification of fluorescence intensity of the nuclear signal of the endothelial-specific β-catenin chromobody was normalized to *Tg(kdrl:*NLS-mcherry*)* expression. A 4-hydroxytamoxifen stock was prepared at 25 mM in DMSO and used at 5 μM to treat embryos.

### Randomization and blinding procedures

After selecting embryos or larvae for the relevant fluorescence signal, they were allocated randomly to different experimental groups. Animals were collected, grown or processed in the same dish or tube for the imaging and analysis. Whenever possible, the investigators were unaware of allocation during experiments, data collection and analysis.

### Statistical analysis

Statistical analysis was performed using the GraphPad software. Values are presented as mean±s.d. *P*-values were calculated using an unpaired two-tailed Student's *t*-test and a one-way ANOVA (post hoc Tukey's test) for GSK3β inhibitor treatment.

## Supplementary Material

10.1242/develop.202122_sup1 Supplementary information
