# Supplementary material for: A β-catenin chromobody-based probe highlights endothelial maturation during vascular morphogenesis in vivo
Source: Development. 2024 Jun 7;151(11):dev202122. doi: 10.1242/dev.202122 (PMC11190570; doi:10.1242/dev.202122)
Supplement: Supplementary information [file develop-151-202122-s1.pdf]

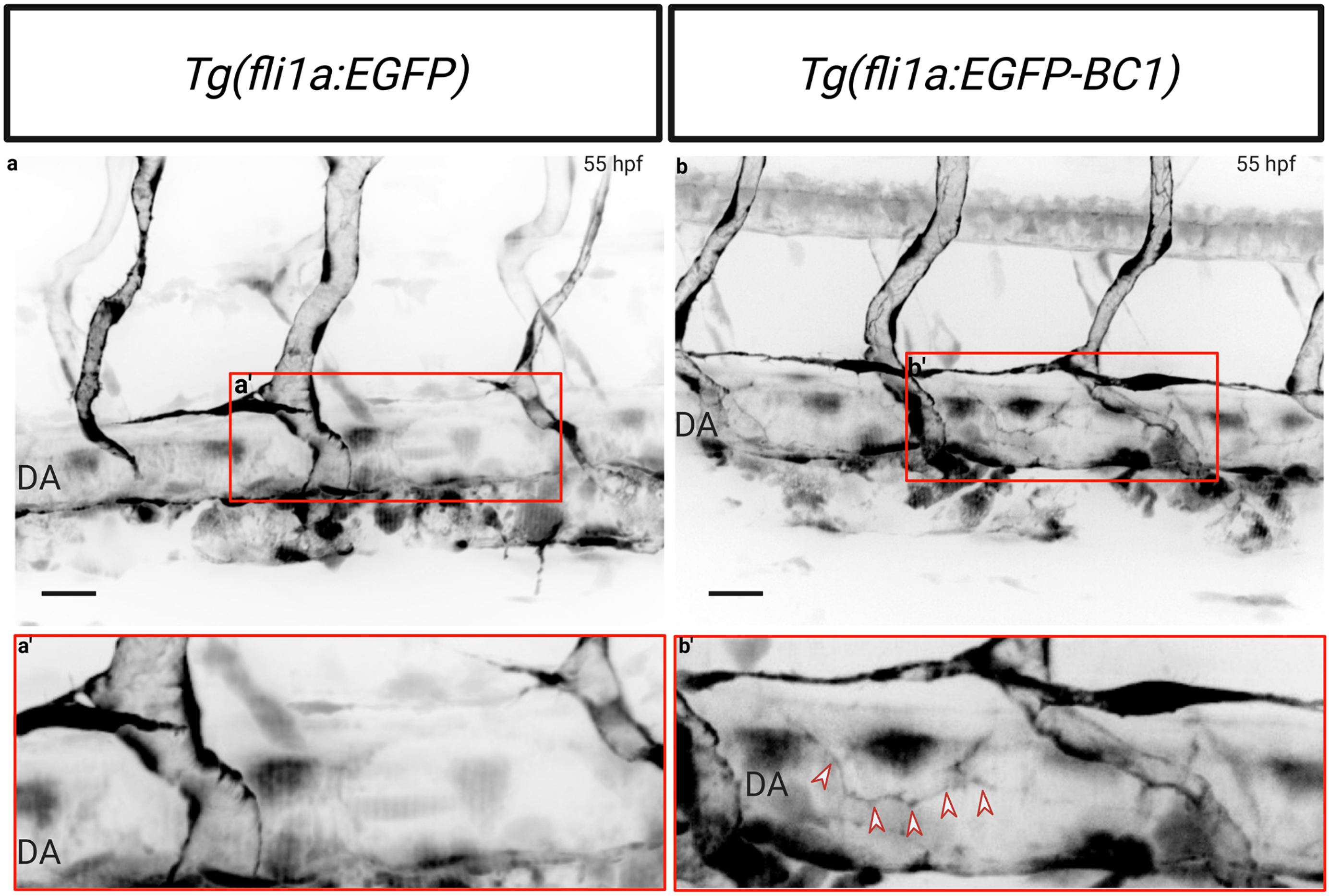

Fig. S1. Comparison between *Tg(fli1a:*EGFP*)* and *Tg(fli1a:*EGFP-BC1*)* expression. a-b) Trunk vasculature of *Tg(fli1a:EGFP)* and *Tg(fli1a:EGFP-BC1)* embryos at 55 hpf. The chromobody expression pattern differs from that of EGFP alone. a'-b') Insets show high magnification images of the dorsal aorta. ß-catenin chromobody localization can be observed at endothelial cell-cell junctions (arrowheads). Scale bars= 40 µm.

Development • Supplementary information

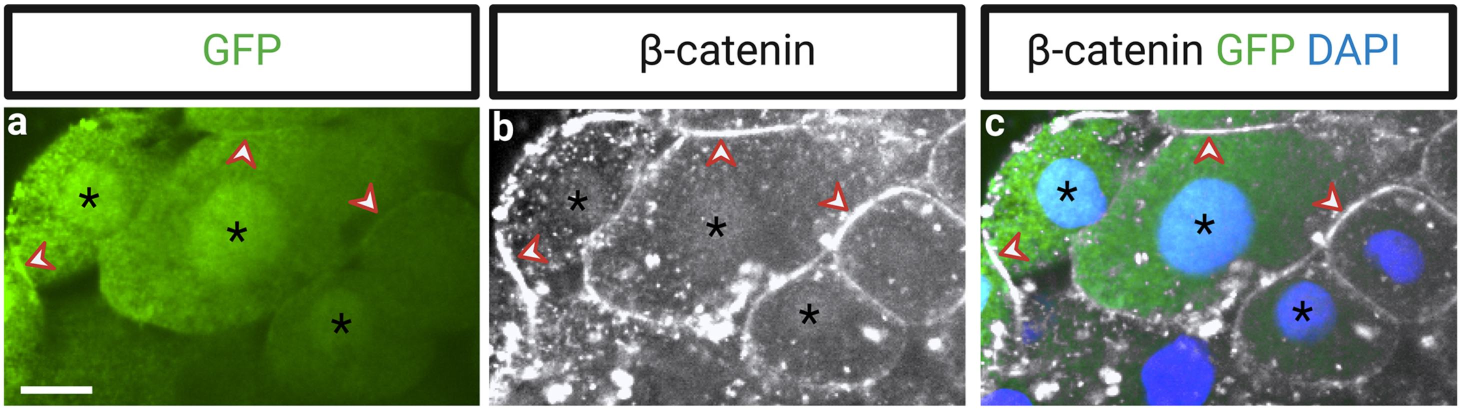

Fig. S2. ß-catenin chromobody expression in blastoderm nuclei and junctions. a-c) Confocal images of immunostaining (GFP, green; ß-catenin, white) and counterstaining for DNA (DAPI, blue) of 512-cell stage embryos injected at the one-cell stage with *EGFP-BC1* mRNA. ß-catenin immunostaining coincides with chromobody localization at blastoderm cell-cell junctions and in the nucleus (arrowheads point to cell-cell junctions; black asterisks mark positive nuclei). Scale bar= 10 µm.

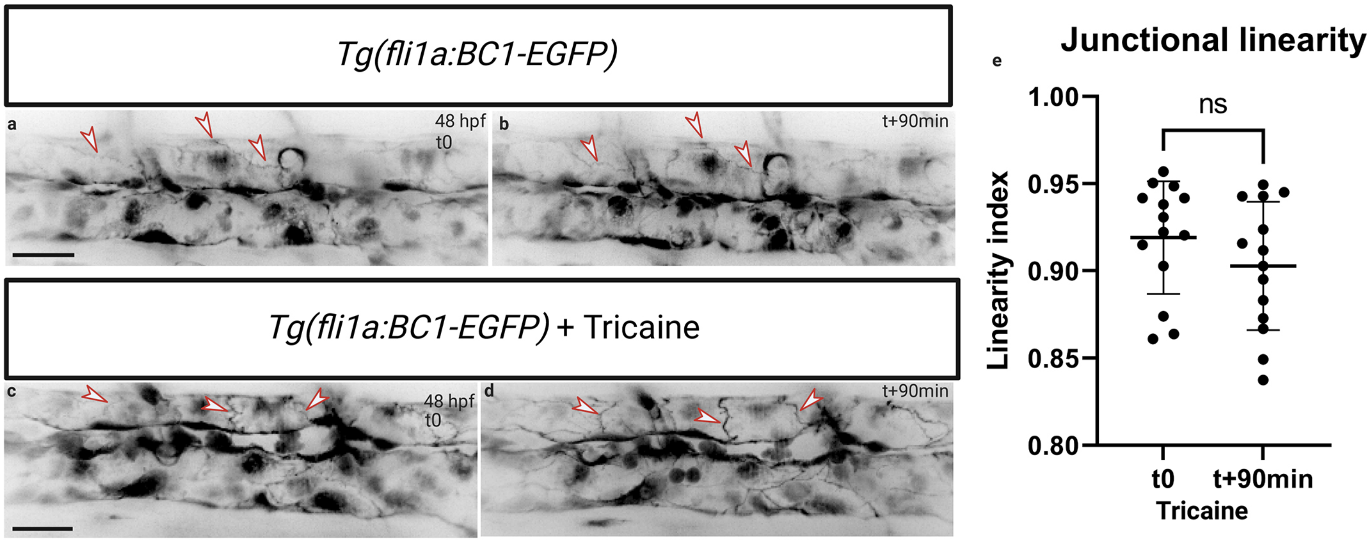

Fig. S3. Acute loss of blood flow does not alter the localization of the endothelial-specific ß-catenin chromobody at endothelial membranes. a-b) Trunk vasculature of a 48 hpf *Tg(fli1a:EGFP-BC1)* embryo at t0 (a) and t+90 min without tricaine treatment (b). c-d) Trunk vasculature of a 48 hpf *Tg(fli1a:EGFP-BC1)* embryo at t0 (c) and t+90 min with tricaine treatment (d). e) Junctional linearity index between two time points, at the start of tricaine treatment (t0) and after 90 minutes (t+90min) (n=14 junctions from n= 4 embryos). Tricaine treatment does not appear to alter junctional linearity, although it does appear to increase chromobody expression at endothelial membranes. Arrowheads point to endothelial cell-cell junctions. Error bars represent mean ± s.d.; *p* value calculated using Student's *t*-test. Scale bars= 40 µm.

Development • Supplementary information

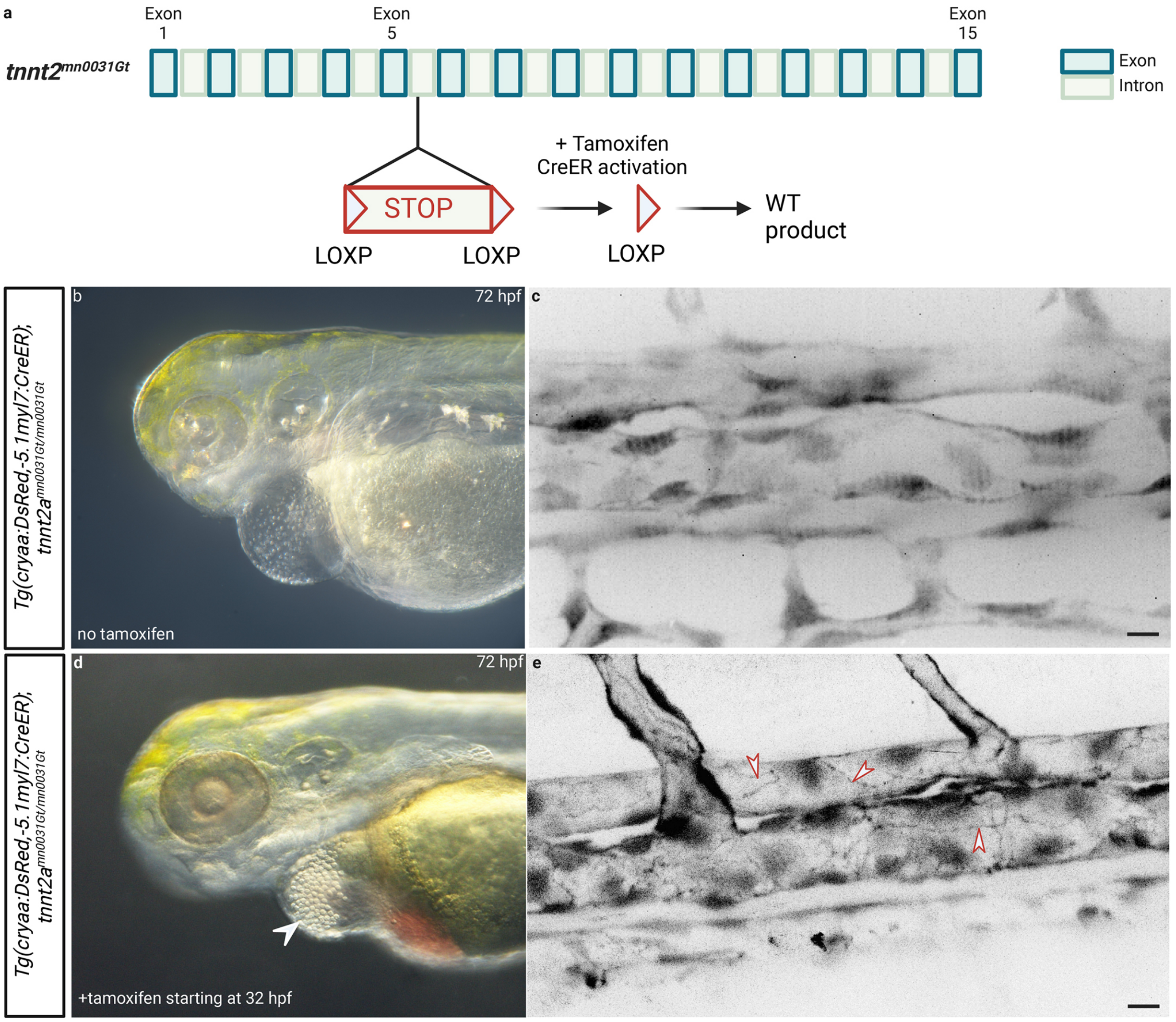

Fig. S4. Rescue of *tnnt2a* mutants by Cre-mediated recombination in *myl7* expressing cells. a) Schematic of the *tnnt2a* locus in the *mn0031* gene-trap line and the effect of tamoxifen treatment to trigger CreER activation. b) Brightfield lateral view of a 72 hpf *Tg(cryaa:DsRed,-5.1myl7:CreER)*; *tnnt2a*$^{-/-}$ zebrafish without tamoxifen treatment. c) Trunk vasculature of a 72 hpf *Tg(fli1a:EGFP-BC1)*; *Tg(cryaa:DsRed,-5.1myl7:CreER)*; *tnnt2a*$^{-/-}$ zebrafish. d) Brightfield lateral view of a 72 hpf *Tg(cryaa:DsRed,-5.1myl7:CreER)*; *tnnt2a*$^{-/-}$ zebrafish after tamoxifen treatment starting at 32 hpf. Rescued *tnnt2a* mutants display reduced pericardial edema (arrowhead) as well as some blood flow. e) Trunk vasculature of a 72 hpf *Tg(fli1a:EGFP-BC1)*; *Tg(cryaa:DsRed,-5.1myl7:CreER)*; *tnnt2a*$^{-/-}$ zebrafish (n=10 embryos from 3 independent experiment). Restoration of blood flow in *tnnt2a*$^{-/-}$ zebrafish leads to the localization of the ß-catenin chromobody at endothelial cell-cell junctions (red arrowheads). Scale bar= 40 µm.

Development • Supplementary information

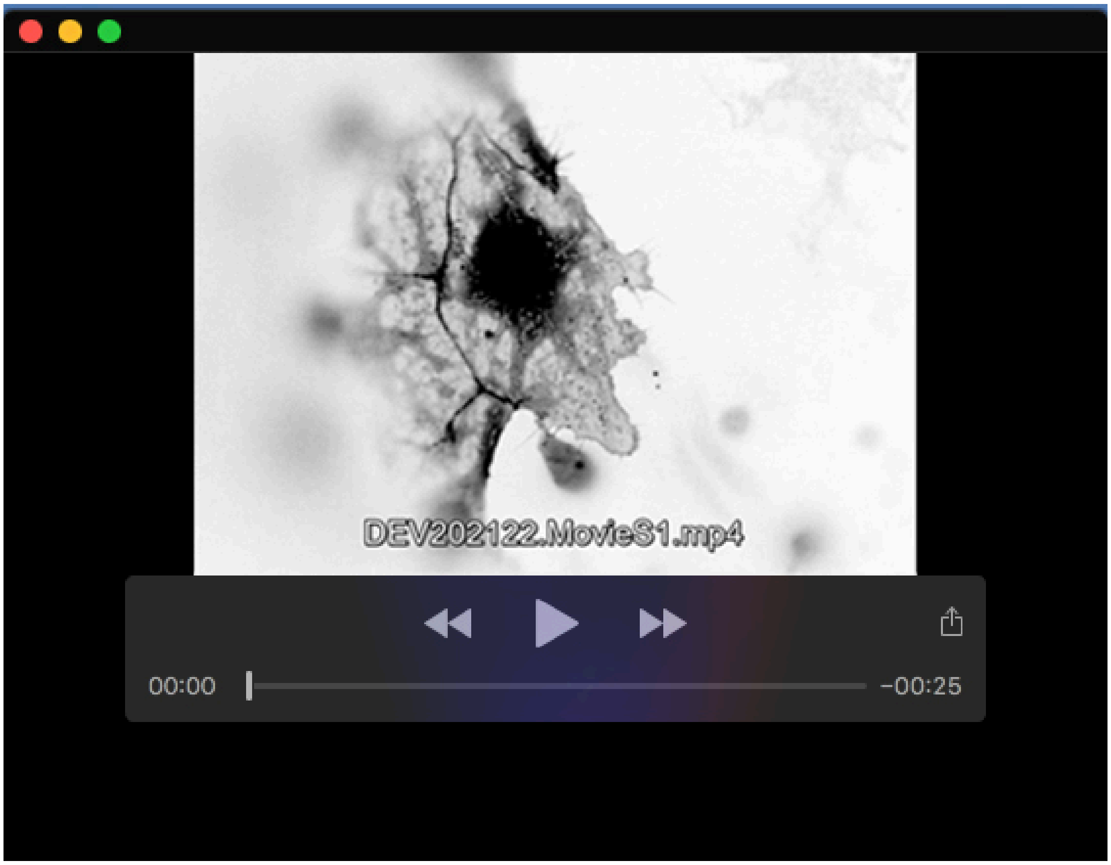

Movie 1. Time-lapse spinning disc confocal microscopy of a single endothelial cell in a *Tg(fli1a:EGFP-BC1)* embryo during the formation of the CCV at 36 hpf (related to Fig.1).

Development • Supplementary information

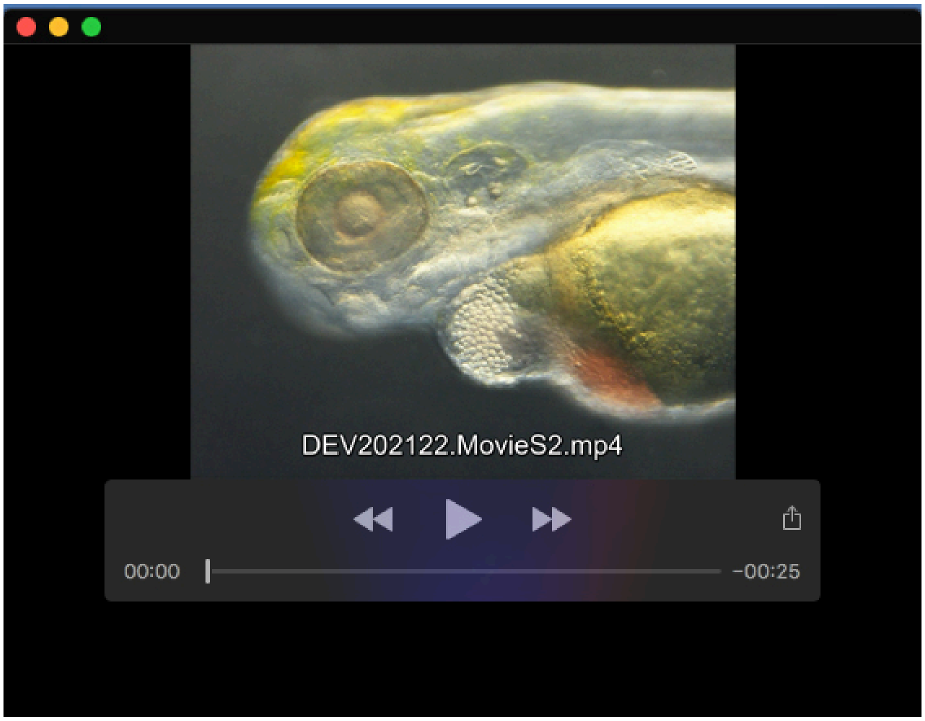

Movie 2. Brightfield lateral view of a 72 hpf *Tg(cryaa:DsRed,-5.1myl7:CreER)*; *tnnt2a*$^{-/-}$ zebrafish after tamoxifen treatment starting at 32 hpf. The rescued *tnnt2a* mutant displays a beating heart and some blood flow (related to Supplemental Fig. 4).

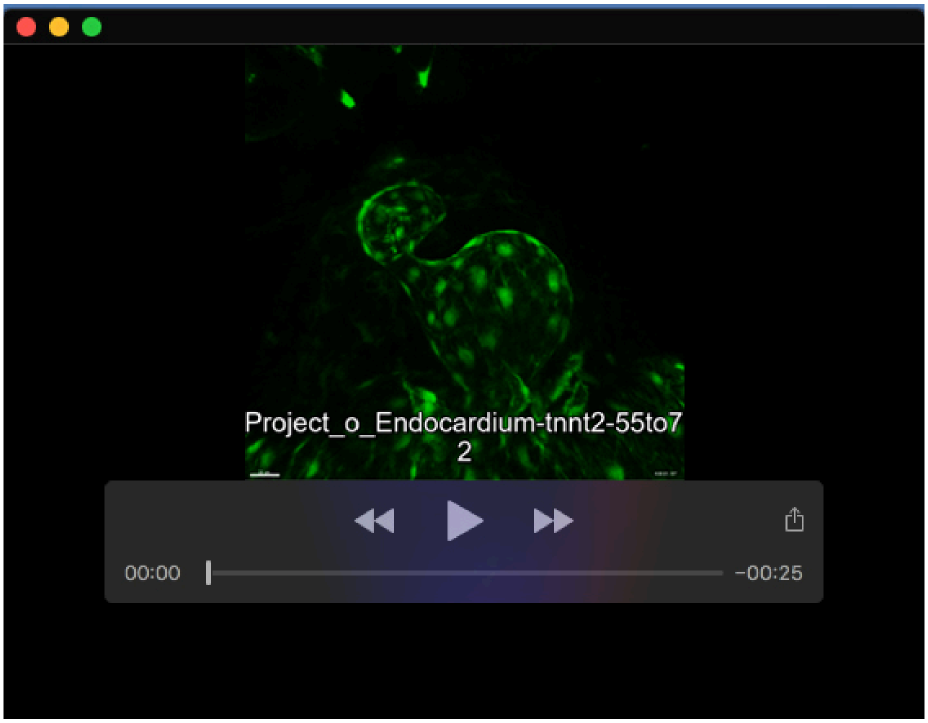

Movie 3. Time-lapse confocal microscopy of cardiac development in a *Tg(fli1a:EGFP-BC1) tnnt2a* mutant from 55 to 72 hpf (related to Fig. 4).

Development • Supplementary information
